# Clinical and laboratory parameters as predictors of long-term outcome according to the etiology of underlying chronic liver disease in patients who underwent liver transplantation for hepatocellular carcinoma treatment

**DOI:** 10.6061/clinics/2020/e1529

**Published:** 2020-05-25

**Authors:** Paulo Henrique Costa Diniz, Serena Dafne do Carmo Silva, Luciana Costa Faria, Paula Vieira Teixeira Vidigal, Teresa Cristina de Abreu Ferrari

**Affiliations:** I Servico de Oncologia, Hospital das Clinicas, Universidade Federal de Minas Gerais, Belo Horizonte, MG, BR; II Departamento de Clinica Medica, Faculdade de Medicina, Universidade Federal de Minas Gerais, Belo Horizonte, MG, BR; III Departamento de Anatomia Patologica e Medicina Legal, Faculdade de Medicina, Universidade Federal de Minas Gerais, Belo Horizonte, MG, BR

**Keywords:** Hepatocellular Carcinoma, Liver Transplantation, Liver Cirrhosis, Recurrence

## Abstract

**OBJECTIVES:**

This study aimed to analyze clinical and laboratory parameters and their association with long-term outcomes in patients who underwent liver transplantation for hepatocellular carcinoma treatment, according to the etiology of the underlying chronic liver disease, in order to identify predictors of response to this therapeutic modality.

**METHODS:**

Demographic, clinical, and laboratory data from a cohort of 134 patients who underwent orthotopic liver transplantation for hepatocellular carcinoma treatment at a referral center in Brazil were retrospectively selected and compared according to the etiologic group of the underlying chronic liver disease. Events, defined as tumor recurrence or death from any cause, and event-free survival were also analyzed, and multivariate analysis was performed.

**RESULTS:**

The etiologies comprised hepatitis C and B virus infection, alcohol abuse, and cryptogenic disorder. Although liver transplantation was performed outside the Milan criteria in 33.3% of the subjects, according to pathologic examination of the explanted liver, the Model for End-Stage Liver Disease score was low (<22) in most patients (70.6%) and recurrence was identified in only 10 (7.9%) patients. Events occurred in 37 patients (28.5%), and the median event-free survival was 75 months (range, 24-116 months). No difference among etiologic groups was found in the parameters analyzed, which were not independently associated with outcome.

**CONCLUSION:**

Clinical and laboratory characteristics according to etiologic groups were not different, which might have led to comparable long-term outcomes among these patient groups and failure to identify predictors that could aid in better selection of subjects for liver transplantation in the management of this cancer.

## INTRODUCTION

Hepatocellular carcinoma (HCC), the fourth leading cause of cancer mortality worldwide, is a complex and poorly understood disease (1,2). It arises in the context of progressive underlying chronic liver disease (CLD) due to etiologies that are very common worldwide, such as hepatitis B (HBV) and C (HCV) virus infection, excessive alcohol consumption, autoimmune hepatitis, nonalcoholic fatty liver disease (NAFLD), inherited metabolic disorders, and other nonidentified etiologies, called cryptogenic etiologies (3).

Only 30-40% of all HCCs are diagnosed at an early stage, for which there are potential curative treatment options, such as liver transplantation (LT) (4). In Europe and the United States, nearly 25-35% of all LTs are performed to treat HCCs (5). In Brazil, where the HCC incidence among patients under surveillance programs is about 2.8% each year (6), there is a report stating a similar proportion - around 33% of LTs (7).

After a period of unrestricted LT indications for treating HCC, the Milan criteria (MC) were introduced in 1996, and are currently a well-established predictor of HCC recurrence (8). These criteria rely on imaging findings, and set restrictive limits based on the size and number of tumors to select candidates for LT. Patients whose tumor burden is within the MC have a 4-year recurrence rate after transplantation of less than 10%, whereas it is about 41% for those with a tumor burden outside the MC (8).

However, the MC have some limitations because the tumor biology, another important determinant of the risk of tumor recurrence after LT, is not taken into account (9). Thus, it is necessary to identify other prognostic and predictive parameters of HCC recurrence. Some studies suggest that pre-LT serum levels of alpha-fetoprotein (AFP), a biomarker used for HCC surveillance and diagnosis, have prognostic utility in monitoring HCC patients treated with LT; however, the cutoff values have not yet been established (9). Moreover, attempts have been made to determine other pathological features and relevant clinic data associated with HCC recurrence, but the results have not yet been validated (10,11).

As HCC is a heterogeneous disorder, with multiple etiologic factors (12), it is possible that there may be differences among the characteristics of the HCC patients as well as long-term outcomes. Therefore, this study aimed to investigate pre-LT clinical and laboratory parameters in patients with HCC related to different etiologies of CLD in a Brazilian population, correlate these findings with the risk of HCC recurrence, and investigate if there is any clinical or laboratory marker that may improve the performance of the MC in predicting HCC recurrence after LT.

## METHODS

### Cohort

Patients with HCC who underwent LT at Hospital das Clínicas, Universidade Federal de Minas Gerais, a Brazilian referral center, between 1998 and 2015, were identified (n=156). The protocol of this institution follows the International Working Party recommendations for HCC diagnosis (13). All patients had archived formalin-fixed, paraffin-embedded tissue of the explanted liver. No histological review was specifically performed for this study, and patients without a definitive diagnosis of HCC (n=3) by histological examination or immunohistochemical analysis were not included in the database.

Demographic and clinical pre-LT data of each patient were retrospectively collected from the medical records. We searched for data regarding sex; age; etiology of the underlying CLD; MELD (Model For End-Stage Liver Disease) and CHILD-Pugh scores - prognostic models that estimate the severity of the underlying liver condition (14,15); number of nodules; size of the greatest nodules; MC determined by pathologic examination of the explanted liver (pathologic MC); diabetes mellitus; AFP levels; hemoglobin levels; international normalized ratio (INR); serum albumin levels; bilirubin levels; liver enzyme levels (aspartate aminotransferase [AST], alanine aminotransferase [ALT], gamma-glutamyltransferase [GGT], alkaline phosphatase [AF]); and lactate dehydrogenase (LDH) levels. As there were only a few cases (n=6) of CLD caused by autoimmune and metabolic disorders, they were not included in the study.

In this study, an event was defined as tumor recurrence or death from any cause. If the patient presented with multiple occurrences, only the first event was counted. Event-free survival (EFS) was defined as the time interval between the LT date and the occurrence of the event or the end of the follow-up period. This information was also obtained from the medical records, but in eight cases, we needed to contact the patient or family by telephone because the patient’s follow-up after LT had been conducted at another institution. The end date of the follow-ups was December 20, 2017.

**Figure 1:**
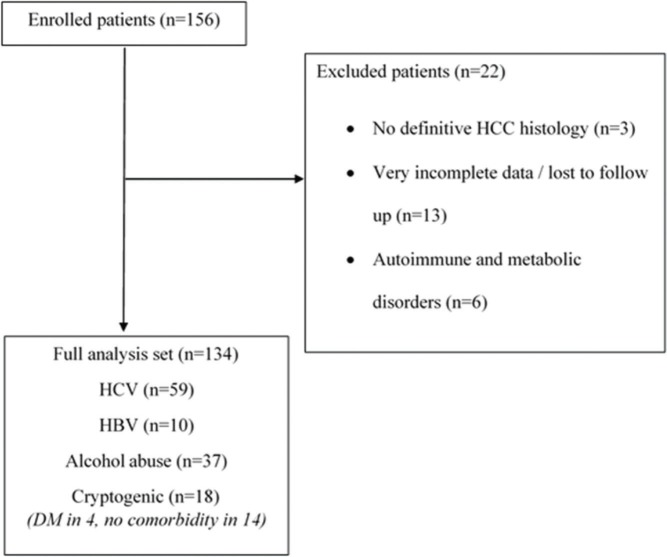
Study diagram flow. HCC: hepatocellular carcinoma; HBV: hepatitis B virus; HCV: hepatitis C virus; DM: diabetes mellitus.

In situations in which data could not be accessed, such as missing information in medical records or loss to follow-up, analyses were performed considering only the available information. Patients with very incomplete data in the medical records of our institution (*i.e.* clinical and laboratorial data and surveillance, simultaneously), whose information could not be recovered from another source (n=13), were excluded (Figure 1).

Before beginning the study, this study was approved by the Ethics Committee of the Universidade Federal de Minas Gerais (CAAE - 44573615.7.0000.5149), and the protocols were in accordance with the ethical guidelines of the 1975 Helsinki Declaration. Written informed consent was obtained from the patients or their relatives. The LTs were orthotopic and exclusively from deceased donors, and the candidates were enrolled in a central waiting list.

### Statistical analysis

Descriptive statistics were used to summarize the data. A normality test (Shapiro-Wilk) was performed for each continuous variable. Categorical data are presented as numbers and percentages, and continuous data are expressed as medians and interquartile ranges. Categorical variables were compared between groups of the four most common etiologies of underlying CLD (alcoholic, B or C viral hepatitis, or cryptogenic) using the chi-square test or Fisher’s exact test, as appropriate. For the comparison of continuous data, we used the Mann-Whitney U test or the Kruskal-Wallis test, as appropriate. In multiple comparisons, Bonferroni correction was applied, when necessary.

Kaplan-Meier curves were constructed to estimate the probability of surviving, considering the four etiological groups and the two main categories (viral and non-viral). The log-rank test was used to compare the curves. A Cox regression model was performed to determine the characteristics that were independently associated with EFS. Furthermore, we assessed the associations between these clinical and laboratory parameters and HCC recurrence or death unrelated to the cancer recurrence, separately, *i.e.*, as two independent outcomes. For this analysis, we used the multivariate Poisson regression model with covariance structure. Clinical and laboratory variables that were associated with the outcome in the univariate analysis (*p*<0.20) were included in the multivariate model. Statistical significance was assumed at *p*<0.05. Statistical analysis was carried out using SPSS software, version 20 (SPSS, Chicago, IL).

## RESULTS

Patients’ demographic and laboratory characteristics according to the CLD etiology are summarized in Table 1. Among the 134 patients analyzed, HCC was related to HCV infection in 59 (61.5%), alcohol abuse in 37 (27.6%), cryptogenic disorder in 18 (13.4%), and HBV infection in 10 (7.5%).

Even though the MC were used to select LT candidates to treat HCC, in 41 of 123 (33.3%) patients, the procedure was performed outside these criteria, considering the pathological examination results of the liver explant. A total of 26/126 (20.6%) patients underwent LT despite having four or more nodules. The median size of the biggest liver nodule was 2.7 cm (range, 2-4 cm). The majority (70.6%) of patients underwent LT with a MELD score below 22, and the cirrhosis was scored CHILD-Pugh C in only 15 of 113 individuals (13.3%). The median AFP serum levels were 12 ng/mL (range, 4.9-58.6 ng/mL). No significant difference concerning AFP levels was found among the four etiologic groups in which the patients were categorized.

Comparing the demographic and laboratory parameters between these groups, the variables were well balanced, apart from sex (*p*=0.02) and hemoglobin (*p*=0.004), AST (*p*=0.001), and ALT (*p*<0.001) levels, as shown in Table 1. HCCs due to alcoholic liver disease were more common in men (97.3% of all cases assigned to this etiology), whereas in women, the main etiology was HCV infection (30.4%). Hemoglobin levels were lower in the alcoholic liver disease group (12.1 g/dL) than in the HCV (13.5 g/dL, *p*=0.001) and HBV groups (14.7 g/dL, *p*=0.018), but severe anemia was not found. Both AST and ALT serum concentrations were lower in the alcoholic liver disease group (52 U/L and 32.5 U/L, respectively) than in the HCV group (91.5 U/L, *p*<0.001 and 75 U/L, *p*<0.001). In addition, ALT levels were lower (39.5 U/L) in the cryptogenic group than in the HCV group (75 U/L, *p*<0.01).

In the HBV group, none of patients analyzed had diabetes, but, despite that, there was no significant difference between the etiologic groups (*p*=0.192) concerning the presence of this condition. Considering all the etiologic groups, diabetes was present in 37 (31.9%) of the patients.

Globally, as shown in Table 2, disease recurrence was identified in 10 (7.9%) of 127 patients, although it may also have occurred in patients who died without observation of recurrence. Clinically evident recurrence, followed by death, was observed in six patients. The event rate was 28.5% (37 recurrences or deaths from any cause in 130 patients analyzed).

Events seemed to be more frequent in the HCV group, in which 20 (54%) recurrences or deaths were observed. Nevertheless, there was no significant difference between the etiologic groups (*p*=0.690). The median follow-up period was 53 months (range 1-97 months), and four patients were lost to follow-up.

**Figure 2:**
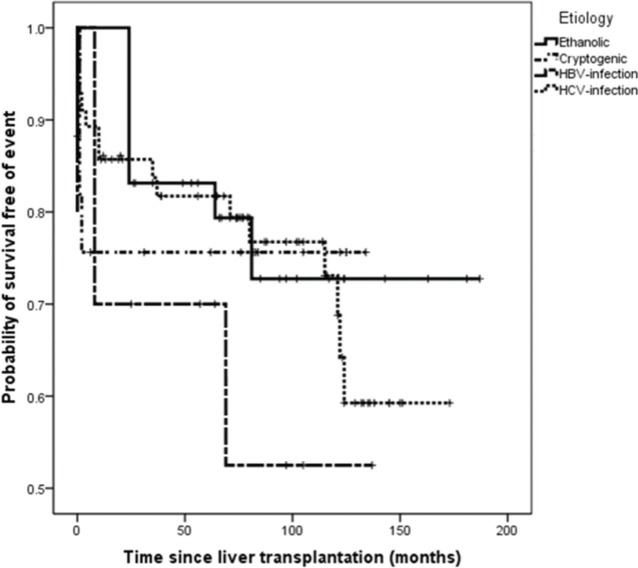
Kaplan-Meier curves of event-free survival in HCC patients according to the etiologic groups of chronic liver disease.

**Figure 3:**
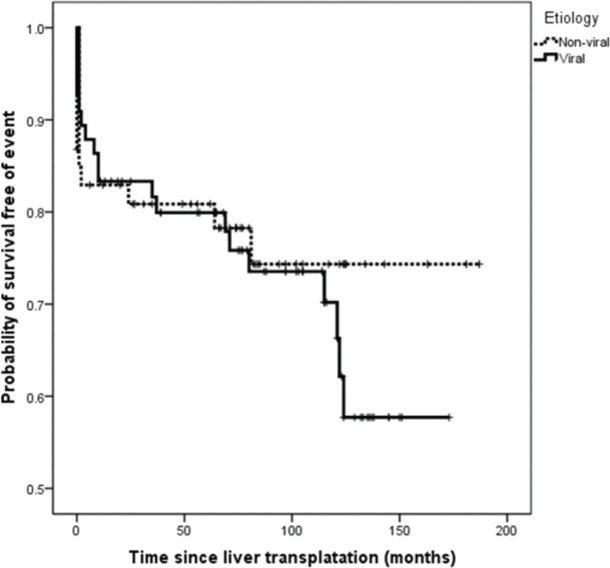
Kaplan-Meier curves of event-free survival in HCC-patients of viral and non-viral etiologic groups of underlying chronic liver disease.

On survival analysis, the median EFS was 75 months (range, 24-116 months). The overall rate of EFS at 12 and 24 months was 83.1% and 82.2%, respectively. Analyzing the four etiologic groups, the Kaplan-Meier curve (Figure 2) showed overlap of the curves (*p*=0.665). The same was seen when the patients were classified in viral and non-viral etiologic groups, as showed in Figure 3.

No demographic or laboratory variables, including the pathologic MC, were found to be related to the risk of event, as demonstrated in both univariate and multivariate analyses (Table 3). Even when HCC recurrence and death unrelated to the cancer recurrence were considered separately as outcomes, no independent association could be demonstrated.

## DISCUSSION

In this study, we found no significant differences in HCC recurrence or mortality profiles among patients who underwent LT to treat HCC in the comparison of the four major etiologic groups underlying CLD. Moreover, similarities in demographic, clinic, and laboratory pre-LT parameters were observed among these four etiologic groups. These similarities, especially the comparable severity of the underlying liver condition among the groups, could be responsible, at least partially, for failing to identify any predictor of recurrence.

Despite refined selection criteria and advances in preoperative staging, recurrence of HCC after LT is still an unsolved issue. There are several studies showing different rates of recurrence (range, 6.4-18.3%) (16-23]. One explanation for this difference could be the time spent on a transplant waiting list. In regions in which this time is short, LT may be performed in candidates with an aggressive tumor biology, among whom high recurrence rates are expected (11). In contrast, in regions in which the waiting time is longer, tumors with an aggressive biology could progress beyond the MC and thus LT is not performed (11). Our findings showed at least 7.5% disease recurrence, which is comparable to the findings of the United Network for Organ Sharing database (23). It is important to highlight that the median follow-up period in our study was only about 53 months.

As recurrence may also have occurred in patients who died without being counted, we defined tumor recurrence or death as events. We found no difference regarding EFS among the etiologic groups. However, when EFS at 12 and 24 months was compared, we observed that most events occurred within the first year of LT, and thereafter, there was a tendency for EFS to plateau. Therefore, it is possible to conclude that many deaths could not be ascribed to HCC recurrence but to non-cancer-related causes, such as LT complications, which are expected to occur within the first few months after the procedure.

Despite the existence of expanded criteria, of which most are not externally validated, the MC are still a useful tool to select HCC patients for LT, and recurrence is more frequent for transplantations outside these criteria (8). In our institution, LT is performed only for patients fulfilling the MC (preoperative imaging with a single lesion <5 cm or up to three lesions ≤3 cm), but our study showed that 33.3% of the patient who underwent LT for HCC treatment were, indeed, outside this criteria based on pathological analysis of explant liver. One possible explanation for this finding is a long time on the waiting list for cadaveric organ donation, during which oligoprogression of the disease could occur. In order to minimize misclassification of tumor extension before LT, the Brazilian government’s rules state that it is necessary to update imaging exams every three months; however, it is known that oligoprogression could occur in tumors with more aggressive behavior, even in this short period. However, the pathological MC were not associated with any differences in EFS in our investigation.

It is worth highlighting that MC are based on imaging methods, and in the present study, the lesions were counted and measured through pathologic examination of the explanted liver. Thus, our patients were classified according to MC, considering the pathological information, by a group of experienced liver pathologists at our institution. It is known that the sensitivity for identifying HCC preoperatively by computed tomography and magnetic resonance imaging has limitations, especially for lesions smaller than 1 cm in diameter (24,25). Therefore, some lesions may have been missed by these imaging methods because of technique-related limitations, measuring errors, or variations in the diameter. These possibilities must have influenced our results. In this setting, a pre-LT imaging review and subsequent analysis would be relevant, but it was not feasible because many of the scans were not performed in our institution, which we consider a limitation of our study.

The MC are considered a good predictor of tumor response to therapy, but they have limitations, and attempts have been made to identify clinical variables that may distinguish patients at higher risk of disease recurrence after LT (9-11). Some studies showed a higher recurrence risk in patients with AFP >200 ng/mL, even within the MC (9,26). In addition to this well-recognized predictor, we analyzed other demographic and laboratory parameters such as age, hemoglobin levels, INR, liver enzymes, and presence of diabetes, and found no predictor of HCC recurrence. Women were more likely to have an HCV etiology, and excessive alcohol intake was the most common etiology in men. Hemoglobin levels were slightly lower in the alcoholic liver disease group and some small differences between groups were observed regarding aminotransferases concentrations, but these factors did not translate into clinical significance.

HCC is a heterogeneous disease and multiple etiologic factors involved can lead to cancer development by different mechanisms (27). Thus, there may be differences in biologic behavior which can translate into clinical outcomes. Retrospective analysis of sorafenib phase III studies in advanced HCC demonstrated that HCV-positive patients had greater benefits from the treatment than did patients with other etiologies (28,29). The phase II study EVOLVE-1 did not demonstrate any benefit of the mTOR inhibitor everolimus on the treatment of advanced HCC, and tumor heterogeneity was suggested as a possible explanation for the poor performance of targeted therapies (30). However, concerning the etiology of the underlying CLD, our study showed no difference in EFS. Furthermore, despite 54% of all events having occurred in the HCV group, the comparison of the event frequency among the groups was not statistically significant, even when grouped as viral and non-viral etiologies.

One important aspect is that our study was conducted in patients who were candidates for LT, which means that the disease was not as advanced as in the above-mentioned studies. Therefore, it is possible that different biological behavior could not translate into clinical outcomes in early disease stages. In 2007, a study conducted in patients with a similar profile showed that cryptogenic cirrhosis was more common in those with recurrent HCC, but this finding was limited because of the small sample size (10).

An important and well-recognized aspect of HCC needs to be mentioned. Liver cirrhosis causes a significant impact on functional liver reserve and is a determinant of the morbidity and mortality associated with HCC (31). Thus, we assessed the severity of the underlying CLD by the CHILD-Pugh score (15), and more than 80% of our patients had scores A or B, indicating that the cirrhosis was not so severe. No significant differences between the etiologic groups were seen. This can explain the relatively long EFS. Moreover, the MELD score (14), a score used to allocate patients in the waiting list for a cadaveric organ according to the severity of cirrhosis, was also analyzed. A large majority of the patients had scores ≤22 and no differences between the groups were noted. In the HBV group, only five (55.6%) of nine patients had scores below this level, suggesting more severe liver dysfunction in this group; however, it included a limited number of patients and the comparison with the other etiologic groups did not reach statistical significance. In Brazil, candidates for LT for HCC treatment receive a special MELD score (7); thus, this is not a good scenario to assess the severity of the underlying CLD.

Despite being a single-center, retrospective study that may harbor selection and information bias, this is an important cohort in which long-term outcomes of LT for the treatment of HCC in a poor-income country were evaluated. As geographic variation in biologic and molecular behaviors of HCC, as well as in the etiology of the underlying CLD, may exist, studies like this are important in elucidating the complexity of the disease. Considering patients who underwent LT, future studies correlating clinical and laboratory parameters with molecular features are possible because the patients’ tumor specimens are available, unlike the case in many HCC patients whose diagnosis is based on non-histological criteria.

This study did not aim to perform a complete histological review of the explanted livers. However, available evidence suggests that histopathological features, such as grade of differentiation and histological type, are relevant in predicting HCC growth rate and its biological behavior (32). In this context, less differentiated tumors and the non-trabecular type (acinar and solid patterns) have been demonstrated to be associated with a higher tumor growth rate and a higher risk of metastasis (33,34). Likewise, the presence of microvascular invasion has been considered a reliable predictor of tumor recurrence (34). Moreover, the relationship between tumor and underlying CLD may also provide important information concerning prognosis; for instance, the presence of a peritumoral capsule is associated with a better outcome after hepatic resection (35,36). Thus, histopathological analysis could identify relevant associations with HCC outcome and should be investigated in further studies.

Another limitation of our investigation is the reduced number of patients in the HBV group. Since the implementation of HBV vaccination by the Brazilian public health system, as well as improved access to antiretroviral treatment, HBV infections have decreased in our country (37). Finally, HCC due to NAFLD was not considered as a separate group but is probably included in the cryptogenic etiology, despite diabetes prevalence not being higher in this group. This condition was only recently recognized as an etiology of HCC, and the exact prevalence of HCC in cirrhotic NAFLD remains unknown (38).

Although the multiple etiologies of CLD and HCC heterogeneity may be determinant factors of response to LT for HCC treatment, our results did not demonstrate any difference regarding the etiology of the CLD and EFS in HCC patients treated with LT. More studies are needed to identify biomarkers with a predictive value to overcome the limitations of MC in predicting outcome and to contribute to appropriate selection of HCC patients for this modality of treatment.

## AUTHOR CONTRIBUTIONS

Diniz PHC collected and analyzed the data, and was responsible for the manuscript writing. Silva SDC collected data and revised the manuscript. Faria LC supervised and supported data collection, and was responsible for the manuscript edition and review.Vidigal PVTchecked statistics, revised the manuscript and analyzed the data. Ferrari TCA evaluated the study quality, designed the study steps, revised the manuscript, and was responsible for the language correction.

## Figures and Tables

**Table 1:** Demographic and laboratorial characteristics according to CLD etiology.

		Overall	HCV	HBV	Alcohol	Cryptogenic	*p*-value
N (%)		134 (100.0)	59 (61.5)	10 (7.5)	37 (27.6)	18 (13.4)	
Age (years)€		57.7	57.0	56.5	57.0	62.5	0.066
		(52.0-63.0)	(51.5-61.5)	(40.5-63.5)	(51.5-63.5)	(47.5-66.2)	
Sex£	F	27 (20.1)	21 (35.5)*	1.0 (10.0)	1 (2.7)	4.0 (22.2)	0.020
	M	107 (79.9)	38 (64.5)	9 (90.0)	36 (97.3)*	14.0 (77.8)	
MELD£	≤22	84/119 (70.6)	42 (71.2)	5/9 (55.6)	24/33(72.7)	13 (76.4)	0.682
	23-29	33/119 (27.7)	16 (27.1)	4/9 (44.4)	9/33 (27.3)	4 (23.5)	
	>29	2/119 (1.7)	1 (1.7)	0/9 (0)	0/33 (0)	1 (5.6)	
CHILD£	A	49/113 (43.4)	27/53 (50.9)	6/9 (66.7)	11/34 (32.4)	5/17 (29.4)	0.202
	B	49/113 (43.4)	22/53 (41.5)	2/9 (22.2)	17/34 (50.0)	8/17 (47.1)	
	C	15/113 (13.3)	4/53 (7.5)	1/9 (11.1)	6/34 (17.6)	4/17 (23.5)	
AFP (ng/mL)€		12.1	17.0	19.4	6.1	8.5	0.421
		(4.9-58.6)	(6.7-72.4)	(3.2-694.0)	(4.1-43.7)	(4.6-79.6)	
Number of nodules£	1	63/126 (50.0)	25/58 (43.1)	6 (60.0)	21 (56.8)	8 (44.4)	0.571
	2	28/126 (22.2)	15/58 (25.9)	2 (20.0)	5 (13.5)	6 (33.3)	
	3	9/126 (7.1)	6/58 (10.3)	0 (0.0)	3 (8.1)	0 (0.0)	
	>3	26/126 (20.6)	13/58 (20.7)	2 (20.0)	8 (21.6)	4 (22.4)	
Size biggest nodule (cm)€		2.7	3.0	2.8	2.5	3.2	0.411
		(2.0-4.0)	(2.05-3.85)	(1.75-4.25)	(1.5-3.3)	(1.0-5.0)	
MC£	Yes	82/123 (66.7)	38/58 (65.6)	7 (70.0)	26 (70.3)	11 (61.1)	0.890
	No	41/123 (33.3)	20/58 (34.4)	3 (30.0)	11 (29.7)	7 (38.9)	
Hb (g/dL)€		13.0	13.5	14.7	12.1*	12.4	0.004
		(11.9-14.4)	(12.7-14.6)	(12.8-15.7)	(11.3-13.2)	(10.7-14.4)	
AST (U/L)€		71.0	91.5	50.0	52.0*	54.5	0.001
		(45.0-104.0)	(58.5-138.2)	(41.0-80.5)	(33.5-78.8)	(41.8-91.8)	
ALT (U/L)€		55.0	75.0	51.0	32.5*	39.5*	<0.001
		(32.7-82.7)	(54.0-102.5)	(42.0-70.5)	(24.0-55.0)	(29.8-61.3)	
Alkaline phosphatase (U/L)€		152.5	140.0	174.0	153.0	206.5	0.247
		(104.0- 234.0)	(101.5-213.5)	(77.0-283.0)	(106.2-253.7)	(129.2-283.5)	
LDH (U/L)€		436.0	441.5	410.0	426.0	495.0	0.803
		(324.0-586.0)	(327.7-618.2)	(316.0-736.0)	(300.0-566.0)	(35.05-660.0)	
Diabetes£	Não	79/116 (68.1)	39 (66.1)	7/7 (100.0)	20/33 (60.6)	13/17 (76.5)	0.192
	Sim	37/116 (31.9)	20 (33.9)	0/7 (0.0)	13/33 (39.4)	4/17 (23.5)	

Data are expressed as absolute numbers (percentage) or median (interquartile range). Number of patients with the characteristic/number for whom the information was available.

CLD: chronic liver disease; HBV: hepatitis B virus; HCV: hepatitis C virus; F: Female; M: Male; MELD: Model for End-Stage Liver Disease; CHILD: CHILD-Pugh score; MC: Milan criteria; Hb: Hemoglobin; AFP: alpha-fetoprotein; AST: aspartate aminotransferase; ALT: alanine aminotransferase; LDH: lactate dehydrogenase.

* Refers to statistically significant difference.

£ Exact Fischer test

€ Kruskal-Wallis test.

**Table 2:** Clinical outcomes according to chronic liver disease etiology.

	All etiologies	HCV	HBV	Alcoholic	Cryptogenic	p-value
Recurrence	10/127 (7.9%)	8/63 (12.7%)	1/10 (10.0%)	1/36 (2.8%)	0/18 (0.0%)	0.173
Event	37/130 (28.5%)	20/65 (30.8%)	4/10 (40.0%)	9/37 (24.3%)	4/18 (22.2%)	0.690
EFS	75 (24.0-116.0)	87.5 (35.5-122.8)	60.5 (6.0-99.0)	69 (26.2-96.2)	76 (1.5-113.5)	0.228

Data are expressed as absolute numbers (percentage) and median (interquartile range).

Number of patients with analyzed outcome/number for whom the information was available.

HBV: hepatitis B virus; HCV: hepatitis C virus; EFS: event-free survival (months).

Fisher’s exact test was used.

**Table 3:** Association between clinical and laboratorial parameters and EFS survival.

	Univariate analysis		Multivariate analysis	
	HR (95% CI)	*p*-value	HR (95% CI)	*p*-value
Hb	0.97 (0.93-1.01)	0.241	--	--
LDH	1.00 (0.99-1.01)	0.090	1.00 (0.99-1.01)	0.168
AST	1.00 (0.99-1.03)	0.124	1.00 (1.00-1.03)	0.180
MELD	0.94 (0.77-1.15)	0.593	--	--
MC	1.17 (0.95-1.44)	0.124	1.23 (0.97-1.55)	0.070
Etiology	0.91 (0.76-1.08)	0.278	--	--

HR: hazard ratio; CI: confidence interval; Hb: hemoglobin; LDH: lactate dehydrogenase; AST: aspartate aminotransferase; MELD: Model for End-Stage Liver Disease; MC: Milan criteria; EFS: event-free survival.
